# Bioavailability and health risk of pollutants around a controlled landfill in Morocco: Synergistic effects of landfilling and intensive agriculture

**DOI:** 10.1016/j.heliyon.2023.e23729

**Published:** 2023-12-19

**Authors:** Hamza El Fadili, Mohammed Ben Ali, Md Naimur Rahman, Mohammed El Mahi, El Mostapha Lotfi, Sami Louki

**Affiliations:** a Laboratory of Spectroscopy, Molecular Modeling, Materials, Nanomaterials, Water and Environment, Materials for Environment Team, ENSAM, Mohammed V University in Rabat, Morocco; b Center for Archaeological Studies, University of Liberal Arts Bangladesh, Dhaka, Bangladesh

**Keywords:** Available fraction, Heavy metals, Human health risk, Montecarlo simulation, Sensitivity analysis, Soil pollution indices

## Abstract

Toxic contamination of agricultural soils by trace metal(oid)s can pose detrimental effects on human health and agroecological systems. In this view, the current research explored total and available metal(oid)s in surface soils and assessed the associated hazards using pollution indices, PMF modeling, PCA, and Montecarlo probabilistic human risk assessment with 10,000 repetitions. The mean concentrations of Cd, Pb, As, Cr, Ni, Cu, Zn, and Fe were 0.89, 24.86, 1.81, 19.10, 25.44, 7.98, 49.12 and 6183.32 mg kg^−1^ dry weight, respectively. These findings highlighted that the concentration of pollutants exceeded the values measured in the geochemical background. Soil enrichment by heavy metal (oid)s was confirmed by analyzing available fractions using DTPA ,CaCl_2_ and enrichment factor (EF). Additionally, pollution indicators (Igeo, PLI, and PERI) displayed significant contamination levels, with a higher ecological risk. Matrix Factorization (PMF) receptor and multivariate statistical analysis reflected that anthropogenic activities, particularly landfilling and agricultural practices were the main causes of the contamination. Furthermore, probabilistic and deterministic human risk assessments showed that carcinogenic risks exceeded the threshold values (10^−4^) set by the USEPA. Consequently, it is crucial to implement continuous monitoring and supervision of landfill sites to prevent additional pollution. These measures should be integrated into the management plans for waste management.

## 1 Introduction

The remarkable and rapid industrial improvement has resulted in a considerable rise in the quantity of waste produced worldwide. Projections indicate that the quantity of waste is anticipated to surge by 50 %, reaching 3.4 billion tons by the year 2025 [1][2]. As a result, addressing these challenges has emerged as one of the most pressing concerns. Despite observing advancements, landfills, and open dumpsites persist as the predominant locations for waste disposal globally [[3]^4^]. As per the International Solid Waste Association (ISWA), more than 40 % of the globally generated waste is improperly discarded [5].

Inadequate disposal of solid waste presents a serious hazard to the environment and all forms of life through the release of hazardous pollutants, including PCBs, PAHs, and metal (oid)s. These pollutants could be discharged into the surroundings through leachate infiltration or as fugitive emissions. Therefore, assessing contamination around landfills and open dumpsites is crucial to evaluate their potential risks throughout all stages of exploitation.

As already mentioned, trace metal (oid)s (TMs) are hazardous elements commonly derived from various human activities (e.g., overutilization of fertilizers and pesticides in agricultural practices, industrial effluents, landfilling practices, and mining activities) [6,[7], [8]]. Unlike many organic pollutants, these substances are widely recognized for their toxicity to human beings, swift biological accumulation, and resistance to biological or chemical degradation, leading to their persistence in the aquatic and terrestrial environment for extended periods [[9], [10], [11], [12]]. Trace metal(oid)s have the potential to disrupt the endocrine system, giving rise to severe health risks for receptors upon contact with contaminated soil through diverse exposure pathways, including inhalation, skin contact, ingestion, and transfer within the soil-food chain. These deleterious compounds can exert both carcinogenic and non-cancerous effects. For example, exposure to Pb and Cd has been linked to renal failure and neurological damage. Moreover, these elements can detrimentally influence soil fertility, and crop production [13,14]. Once trace metal(oid)s (TMs) are present and accumulate in soils, their removal can be a challenging and costly endeavor from a technical standpoint [15].

Given these hazards, there has been a growing interest and numerous efforts undertaken by scientists and stakeholders worldwide to evaluate the environmental occurrence and fate of these hazardous elements, aiming to mitigate their harmful effects and adverse health consequences. As a result, stringent regulations and standards have been established to ensure the safe disposal of waste and the use of manure and fertilizers containing trace metal(oid)s [16].

Assessing the status of trace metal(oid)s in agricultural soil is of utmost importance to ensure better management of soil quality and develop sustainable policies [17]. For this reason, Nemerow pollution (PIN), pollution load (PLI), and enrichment factor (EF) are essential indicators employed for assessing soil contamination and enrichment level by TMs [7], these indicators provide a more comprehensive approach and offer a more robust and systematic way to identify and quantify the impact of human-induced pollution on soil, providing valuable insights for environmental management and remediation efforts.

Previous research has predominantly focused on applying correlation analysis, and multivariate statistics to highlight the sources of TMs in soils and explore their relationships. However, these methods offer only qualitative information regarding the sources of pollution, without quantifying the contribution of each source to the concentration of each metal(oid). To address this limitation, the positive matrix factorization (PMF) technique was introduced by the EPA. This method has been widely adopted in recent studies [16,[18], [19], [20]] and has demonstrated its importance in the analysis of pollutant apportionment. By employing PMF, researchers can quantitatively determine the impact of each pollution source on the measured concentrations of trace metal (oid)s in examined sites, enabling more precise and informed decision-making in environmental management and policy formulation.

For instance, based on a per capita daily footprint of 0.76 kg, an estimated annual generation of approximately 7 million tons of waste is observed in Morocco, and most of them are discarded in landfills without any treatment. There are more than 337 operated landfills of which only 16 are equipped with leachate collection and treatment systems [21]. Factors such as cultural beliefs, lack of infrastructure, inadequately trained humans, and other logistics constraints have been highlighted as the main causes behind the current waste management situation.

Indeed, despite numerous studies on TMs in agricultural soils, there is still a gap in our understanding of the health risks linked to these contaminants in the vicinity of landfills in Morocco. Most previous research has not adequately considered the potential human risk of trace metals (TMs). Therefore, it is essential to address this gap by evaluating the bioavailability, and health risks of TMs in the surface soils by taking the Oum Azza region as a case study.

Hence, the current paper aims; (a) to examine the level and spatial distribution of TMs in agricultural soils; (b) to evaluate the potential impact on human health; and (c) to discern the contributions of various pollution sources behind the measured concentrations of trace metal(oid)s in the investigated soil, using the Positive Matrix Factorization (PMF) model and Principal Component Analysis (PCA). The findings are intended to serve as a reference for future research focusing on trace metal (oid)s in landfills with similar characteristics. By achieving these objectives, the research aimed to contribute to the better understanding and management of trace metal (oid)s pollution.

## 2 Materials and methods

### 2.1 Study area

**Fig. 1:**
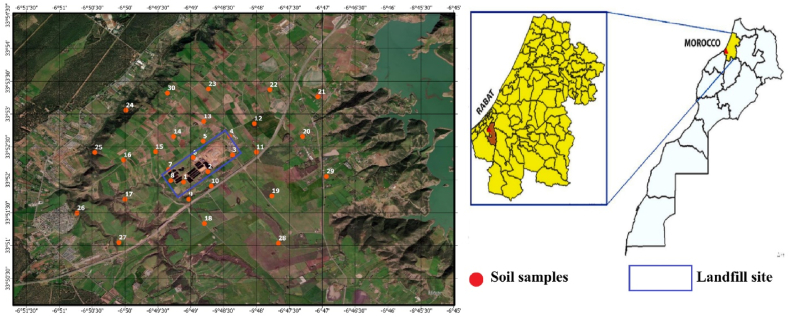
Study area map showing examined soils sampling around Oum Azza site, Morocco.

The examined area is situated in the municipality of Oum Azza (33°52′50.87"N; 6°47′8.55"O) in the northwest region of central Morocco. Between 1994 and 2004, the population of the municipality increased from 8204 to 10530 inhabitants. The area and its surroundings are characterized by the presence of one of the biggest Moroccan landfills for the disposal of 2100 tons per day and intense agricultural activities[22]. The study area falls under arid regions with an annual average precipitation of 548 mm and an average temperature ranging from 7.6 to 28.1 C, with the highest temperature in August and the lowest in January. The hydrogeological and geological conditions are presented in Supplementary materials. The location map of the examined samples is presented in Fig. 1.

### 2.2 Collection and analysis of examined samples

To evaluate the risks of metal (oid)s due to the leakage of leachate and the intensive use of pesticides and fertilizers, 30 surface soil samples were gathered using a clean stainless-steel trowel. At each sampling site, 4 subsamples within a square of 1 m^2^ Samples were gathered and meticulously blended to acquire a representative composite soil. Subsequently, soils were carefully placed into sanitized polyethylene bags and promptly transported to the laboratory in an ice box for analysis.

Upon reaching the laboratory, the examined samples were air-dried, then they were sifted through a 2-mm sieve using vibrating stainless sieves and pulverized manually. For determination of the total fraction of heavy metal(oid)s, the finely powdered samples were subjected to a wet digestion method using Aqua Regia [23,24].

The bioavailable fraction was determined by mixing 10 g of examined soil samples with 20 ml of 0.01 CaCl_2_ for Pb and 0.005 DTPA (diethylene triamine penta-acetic acid) for other pollutants [21]. The digested solution underwent filtration using a Whatman No.42 filter paper, followed by dilution to the desired volume using ultrapure water.

The analysis of metal contents in the solution was carried out using an inductively coupled plasma-atomic emission spectrometer (ICP-AES) as described in our previous paper [4]. To ensure precision, the analyses were conducted in triplicate. Results were considered valid and included in the present paper only if the relative standard deviation (RSD) for three replicates of each sample was less than 5 %.

### 2.3 Soil pollution assessment

To gain insight into the pollution level in the examined area. Different factors and indicators, including the Geo-accumulation index, Nemerow index, Enrichment factor, and Pollution load index, were simultaneously computed for various sampling sites and metal (oid)s. The grading system employed for the analyzed indices is illustrated in Table 1.

**Table 1:** pollution and ecological risk indices.

Class	Igeo	PLI	PERI	EF	PIN
1	Igeo ≤0 unpolluted	PLI <0 low level	≤150 Low risk	EF ≤ 1 no enrichment	PI ≤ 0.7 secure (no impact)
2	0 < I_geo_ < 1 unpolluted to moderately polluted	1 < PLI <2 moderate level	150 < PER ≤300 Moderate risk	1 < EF ≤ 3 minor enrichment	0.7 < PI ≤ 1 alert (very small impact)
3	1 < I_geo_ < 2 moderately polluted	2 < PLI <3 High level	300 < PER <600 Considerable risk	3 < EF ≤ 5 moderate enrichment	1 < PI ≤ 2 slight (lesser impact)
4	2 < I_geo_ < 3 moderately to heavily polluted	PLI >3 Extremely high level	≥600 High risk	5 < EF ≤ 10 moderate to severe enrichment	2 < PI ≤ 3 moderate (medium impact)
5	3 < I_geo_ < 4 heavily polluted			10 < EF ≤ 25 severe enrichment	PI > 3 heavy impact
6	4 < I_geo_ < 5 heavily polluted-extremely polluted			25 < EF ≤ 50 very severe enrichment	
7	I_geo_ ≥ 5 Extremely polluted			EF > 50 extremely severe enrichment	

For the computation of pollution indices, uncontaminated local soil was gathered from a site situated 3 km away from the examined area, serving as the lithogenic reference. The amount of metal (oid)s at this site is: Pb (9.5), Cd (0.3), Cu (3.5), Zn (26.5), Ni (14.3), Cr (12), As (0.625), and Fe (6054.5) in mg⋅kg^−1^ dw.

#### 2.3.1 Geo-accumulation index (Igeo)

Igeo was introduced as shown in equation (1), to assess the pollution status in the examined soils [21].

(1) $$\mathrm{Igeo}=\log_{2}\;\left[\text{Cn}∕1.5\text{Bn}\right]$$

where Cn is the concentration of trace metal (oid) n in the soil, 1.5 is a correction factor that enables the correction of the variability due to the lithogenic influences and Bn is the reference value for each metal (oid).

#### 2.3.2 Pollution load indice (PLI)

PLI integrates Cf values of all measured trace metal (oid)s and enables the evaluation of the soil pollution at each examined site [9]. PLI is calculated as the geometric mean of Cf based on the following equation:

(2) $$\mathrm{PLI}={\left[Cf_{1}\times Cf_{2}\times Cf_{3}\times Cf_{4}\times \cdots \times Cf_{n}\right]}^{1/n}$$

#### 2.3.3 Nemerow index method (PIN)

The Nemerow was also employed in the current study, in order to conclude an overall about the degree of poly-metallic soil contamination, using the following equations:

(3) $$Pi=\frac{C_{i}}{S_{i}}$$

(4) $$PIN=\sqrt{\frac{{\left(\frac{1}{n}\sum_{i=1}^{n}\;PI_{i}\right)}^{2}+{\left[\max\left(PI\right)\right]}^{2}}{2}}$$

where Pi is the single pollution index of metal (i) in the soil, C_i_ and S_i_ are the measured and standard value of each metal (oid) (i), respectively. WHO guidelines for soils quality are selected as the control standard for soil contamination.

#### 2.3.4 Enrichment factor (EF)

To evaluate comprehensively and distinguish between geogenic and anthropogenic sources of pollutants in the examined soils. The EF is computed through the normalization of metal (oid) concentration in the sample with respect to the concentration of a reference element, using the following equation:

(5) $$\text{EF}=\frac{\left(\text{Cn}/\mathrm{Cref}\right)\;\text{Sample}\;}{\left(\text{Cn}/\mathrm{Cref}\right)\;\text{Background}\;}$$

Where Cn is the measured concentration of metal (n) in soil samples and Cref is the same concentration of the chosen reference metal. Fe was chosen as the reference due to its abundance and stability in the examined area.

### 2.4 Ecological risk assessment

Ecological risks index were introduced by Hakanson [21] to evaluate the potential single and multi-element ecological risk based on the toxicity response and examined trace metal (oid)s as shown in equation (6):

(6) $$E_{r}^{i}=T_{r}^{i}\times C_{f}^{i}$$

(7) $$\mathrm{PERI}=\sum_{i=1}^{n}\;E_{r}^{i}=\sum_{i=1}^{n}\;T_{r}^{i}\times C_{f}^{i}$$

where $T_{r}^{i}$ is the toxic-response factor of metal (i), they are 30, 10, 5, 5, 5, 2 and 1 for Cd, As, Cu, Pb, Ni, Cr and Zn, respectively [25]. PERI enables the evaluation of the poly-metallic ecological threat at each sampling site by summing the $E_{r}^{i}$ of every metal using equation (7).

### 2.5 Human health risk appraisal

Health risk due to the accumulation of metal(oid)s in the examined soils is beneficial for assessing the probability of negative health effects on local residents who are exposed to it. Generally speaking, soil with a high level of contaminants may pose significant risks to human beings through inhalation, dermal contact and accidental ingestion pathways. In the current paper, carcinogenic and non-carcinogenic hazards were calculated for residential receptors using the method developed by the United States Environmental Protection Agency (USEPA) for health risk appraisal [26].

The assessment of long-time exposure was performed by assessing the Chronic Daily Intake (CDI: mg⋅kg^−1^/day) for each selected metal(oid), taking into account the potential exposure routes mentioned earlier. The CDI values were determined using the following equations (8), (9), (10):

(8) $${\text{CDI}}_{ing‐soil\;}=\left(\frac{C_{\mathrm{soil}\;}\times {\text{IngR}}_{\mathrm{soil}\;}\times \mathrm{EF}\times \mathrm{ED}\times 10{⌃}^{-6}}{\mathrm{BW}\times \mathrm{AT}}\right)$$

(9) $${\mathrm{CDI}}_{\mathrm{inh}\;}=\left(\frac{C_{\mathrm{soil}\;}\times \mathrm{InhR}\times \mathrm{EF}\times \mathrm{ED}}{\mathrm{BW}\times \mathrm{AT}\times \mathrm{PEF}}\right)$$

(10) $${\text{CDI}}_{\text{der}}=\left(\frac{C_{\mathrm{soil}\;}\times {\text{AF}}_{\mathrm{soil}\;}\times \mathrm{SA}\times \mathrm{ABS}\times \mathrm{EF}\times \mathrm{ED}\times 10{⌃}^{-6}}{\mathrm{BW}\times \mathrm{AT}}\right)$$

The variables and exposure parameters utilized in the calculation are summarized in Table S1 (supplementary material).

#### 2.5.1 Non-carcinogenic risk

In order to compute the non-cancerous risks due to the exposure to trace metal (oid)s, hazard quotient and hazard index were computed using the following equations (11), (12), (13):

(11) $$\mathrm{HQ}=\frac{\text{CDI}}{RfD}$$

(12) $$HI=HQ_{ing}+HQ_{inh}+HQ_{derm}=\frac{C{DI}_{ing}}{RfD_{ing}}+\frac{C{DI}_{inh}}{RfD_{inh}}+\frac{C{DI}_{derm}}{RfD_{derm}}$$

(13) $$THI=\sum_{j=1}^{n}\;HI_{j}$$

HQ is determined by calculating the ratio between CDI and the exposure reference dose (R*f*D) of each pollutant (mg.kg^−1^.Day^−1^). The hazard index (HI) is the sum of HQs that represents the likely NCR induced by each metal, while the total hazard index (THI) measures overall health considering all TMs and various routes of contaminants penetration to human body. THI >1 indicates that metals exposure is likely to have adverse health effects; however, THI <1 indicates that there are no harmful health impacts [26].

#### 2.5.2 Carcinogenic risk

The cancer risk (CR) was calculated to determine the likelihood of cancer developing as a result of lifetime metal exposure. It is worth noting that Carcinogenic health risks were calculated for only Pb, Cd, Cr, Ni, and As for which carcinogenic slope factors (CSF) are available. The Cancer Risk (CR) was estimated using Eqs. (14), (15)

(14) ILCR(IncrementalLifetimeCancerRisk)=CDI×CSF

(15) TCR(Totalcancerrisk)=∑CR(ingestion+inhalation+dermal)

The corresponding values of CSF for the five considered metal (oid)s are presented in Table S1 [27]. Then, the Total cancer risk (TCR) was computed by summing up the ILCR for all metal (oid)s. If the CR and TCR are less than 10^−6^, the carcinogenic risk is considered insignificant or does not affect the human body. While, values above 10^−4^ suggest that there is a carcinogenic risk [21].

#### 2.5.3 Montecarlo simulation-based probabilistic approach & sensitivity analysis

When employing the deterministic method for human health risk appraisal, there are numerous factors that contribute to variability and uncertainty. Variability and uncertainty in input parameters stem from differences in values among individuals and the incomplete understanding of certain parameters. These limitations can lead to underestimating or overestimating human risk [28]. Therefore, it is essential to enhance our understanding of the actual risk to produce reliable outputs and enable decision-makers to make informed choices regarding mitigation measures.

To address these challenges, a Monte Carlo simulation (what-if analysis) was employed to calculate and simulate human risk. This approach helps minimize the uncertainty and variability associated with the deterministic method that relies on single-point variables [18]. Additionally, analysis of sensitivity was utilized to identify the most influential input parameters in Human Health Risk Assessment (HRA). The Crystal Ball software version 11.1.2.4 (Oracle, USA) was used for the simulation, performing 10^4^ iterations with a 95 % confidence level. The probabilistic distribution of the Total Hazard Index (THI) and Total Cancer Risk (TCR) was determined based on the distribution of input variables presented in Table S2, resulting in credible risk outputs. This integrated approach aids in obtaining more robust risk assessments, enhancing the accuracy of results, and enabling better decision-making regarding human health protection measures.

### 2.6 Positive matrix factorization (PMF) model

In order to provide an in-depth understanding regarding the source of TMs in examined agricultural soils. Positive matrix factorization (PMF) was employed. PMF, initially proposed by the USEPA, has been widely utilized as an effective receptor and exploratory model [13,29]. This method enables the quantitative determination of the contribution of each pollution source to the presence of various trace metal (oid)s in the soil. Mathematically it can be formulated using equation (16) presented below:

(16) $$Q=\sum_{i=1}^{m}\;\sum_{j=1}^{n}\;{\left[\frac{x_{ij}-\sum_{k=1}^{p}\;g_{ik}f_{kj}}{u_{ij}}\right]}^{2}=\sum_{i=1}^{m}\;\sum_{j=1}^{n}\;{\left[\frac{e_{ij}}{u_{ij}}\right]}^{2}$$

When the concentration of trace metals (TMs) is equal to or falls below the respective minimum detection limit (MDL), the uncertainty u_ij_ is computed through equation (17):

(17) $$c\leq \text{MDL},u_{\text{ij}}=5/6\times \text{MDL}$$

Otherwise, u_ij_ is calculated using the following formula (equation (18)):

(18) $$c>MDL,u_{ii}=\sqrt{{\left(\;\mathrm{error}\;\mathrm{fraction}\times c\right)}^{2}+MDL^{2}}$$

Minimum detection limit (MDL) values were 0.001 mg kg^−1^ for all metal (oid)s. The calculation was conducted using the EPA PMF 5.0 software.

### 2.7 Statistical analysis

Normality distribution of the collected samples was evaluated using Kolmogorov-Smirnov (K–S) test with a significance level of p < 0.05 was employed. As the majority of the examined variables did not follow normal distribution, a non-parametric test was used to explore possible cause-effect associations among them.

For the exploration of large datasets and the identification of potential sources of contamination (geogenic and/or anthropogenic) as well as determining the number of factors involved, multivariate statistical analyses (MSAs) were employed. Among these techniques, Principal Component Analysis (PCA) is one of the commonly methods in environmental studies. PCA acts as a dimension reduction technique, transforming complex data into a set of variables known as principal components (PCs), which simplifies data interpretation. By applying PCA, researchers can gain valuable insights into the underlying patterns and relationships among the examined samples.

To ensure the adequacy of the data for this analysis, Bartlett's Sphericity and the Kaiser Meyer Olkin (KMO) tests were conducted [21]. Additionally, a Hierarchical Cluster Analysis (HCA) using Ward's method with squared Euclidean distance was performed to assess the effectiveness of the assessment tools in grouping datasets based on the similarities between examined variables.

All statistical calculations used in the current paper were performed using XLSTAT software, and considering a significant level of 0.05.

## 3 Results and discussion

### 3.1 Descriptive statistics of trace metal (oid)sin soils

A summary of the examined metal (oid)s amount from the thirteen examined sites is listed in Table 2. The concentration exhibited significant variation across the sampling sites, as follows (in mg⋅kg^−1^ dw): Cd (0.21–2.93), Pb (12.24–78.97), As (0.52–2.99), Cr (9.71–51.90), Ni (9.64–53.72), Zn (29.20–85.41), and Fe (5.67–6.58 g/kg). Among the detected TMs, the average concentrations of Fe and Zn were found to be the highest, followed by Ni and Pb, and finally Cr, Cu, As, and Cd. Moreover, several TMs (i.e., Pb, Cd, As, Cr, As, and Cu) exhibited abnormal distribution, reflected by high coefficient of variation (CV) values.

**Table 2:** Descriptive statistics of metal (oid)s amount (mg/kg dry weight).

Metal (oid)s	Cd	Pb	As	Cr	Ni	Cu	Zn	Fe
**Minimum**	0.210	12.235	0.520	9.714	9.642	2.758	29.200	5671.500
**Maximum**	2.930	78.968	2.988	51.900	53.728	16.481	85.406	6583.000
**1st Quartile**	0.368	15.457	1.402	12.512	16.225	4.120	36.588	6072.500
**3rd Quartile**	0.929	25.198	2.182	23.625	31.519	11.114	63.193	6309.250
**Mean**	0.889	24.861	1.816	19.096	25.438	7.984	49.126	6183.317
**CV**	76.97 %	59.93 %	35.13 %	51.44 %	45.52 %	51.34 %	31.10 %	3.09 %
**K–S test**	<0.0001	<0.0001	0.050	<0.0001	0.009	0.033	0.026	0.050
**Average background**	0.3	9.5	2.5	12	14.3	3.5	26.5	6054.5

The CV decreased in the following order; Cd (76.97 %) > Pb (59.93 %) > Cr (51.44 %) > Cu (51.34 %) > Ni (45.52 %) > As (35.13 %) > Zn (31.10 %) > Fe (3.09 %). The elevated CV values highlighted that human activities significantly influenced the spatial distribution of pollutants around the study area, consistent with previous outcomes obtained by Islam et al. [30]and Kumar et al. [31]who elucidated this distribution to human activities.

By comparing the mean concentrations of TMs with the geochemical background values, it is clearly observed that the concentrations of Cd, Pb, As, Cr, Ni, Cu, Zn, and Fe were 2.96, 2.62, 2.91, 1.60, 1.78, 2.28, 1.85, and 1.02 times higher, respectively, than those measured in the reference sample. Specifically, around 80 %, 83 %, 87 %, 90 %, 93 %, 93 %, 100 % and 100 % of samples exceeded the corresponding geochemical background value for Cd, Cr, Cu, Ni, As, Fe, Zn, and Pb, respectively.

Thus, local man-made activities including landfills, recycling center, fertilizers,agrochemicals, and traffic emissions, brought a non-negligible amount of TMs into the soils of the examined area.

Notably, the concentration of pollutants was generally higher in the soils from the examined sites, S1∼S4, S6∼S8, S10∼S12, and S14∼S15, marking them as the most polluted compared to other sites. This observation suggests that the study soils have been locally affected by the combined accumulation of hazardous elements originating from municipal waste landfilling and agricultural runoff. Overall, the concentrations of Cd, Cu, Pb, and Zn decreased in line with the distance from the landfill, indicating that landfilling practices are the most substantial source of pollution in the investigated area.

The bioavailable fraction of metals extracted using DTPA and CaCl_2_ for Cd, Pb, As, Cr, Ni, Cu, Zn, and Fe were found to be (in mg⋅kg^−1^ dw): 0.03–0.44, 0.25–9.43, 0.013–0.42, 0.15–3.84, 0.25–2.64, 0.07–3.65, 0.84–3.61 and 19.10–73.25, respectively. While, those of the geochemical background were, as follows: 0.1, 0.035, 0.042, 0.10, 0.23, 0.07, 0.45 and 9.68, respectively. These results indicate that mismanagement of landfilling practices and agricultural activities have significantly contributed to an increase in the extractable fraction of all metal(oid)s compared to the reference values in the decreasing order of: Cd > Pb > As > Cu > Ni > Cr > Zn > Fe. This indicates that the majority of metal(oid)s across the study area exhibit high mobility and bioavailability, which can have implications for potential environmental and human health risks [32].

Even with this redundancy, the above findings demonstrate that the amount of metal (oid)s in the soils is still below the permissible contamination levels established by the WHO as a threshold values [21].

The comparison between the concentration of pollutants in the examined soils and those reported in previous studies from agricultural sites worldwide is presented in Table 3. Overall, it is observed that the amounts of As, Cr, Cu and Pb were notably lower than those measured in Harran Plain in Turkey [25], Isfahan city in Iran [33] and Benguerir in Morocco [21]. However, it was clearly concluded that the concentrations of TMs in our study were higher than those measured in Tunisian and Nigerian surface soils. This variation can be attributed to the spatial heterogeneity in anthropogenic activities in each region. Additionally, soil properties such as organic matter content, pH, and mineralogy can affect the retention and availability of TMs in the soil. Therefore, it is essential to consider these regional and site-specific factors when interpreting and comparing TMs concentrations across different studies [21].

**Table 3:** The comparison of metal (oid)s concentration in examined soils with other agricultural areas in Morocco and worldwide.

Pb	Cd	As	Cr	Ni	Cu	Zn	References
12.23–78.96	0.21–2.93	0.52–2.98	9.71–51.90	9.64–53.72	2.75–16.48	29.20–85.41	This study
2.20–115.52	0.36–6.80		3.73–68.19	1.25–34.20	2.50–54.50	7.92–190.50	[21]
1.8–13.2	0.3–1.2		34.4–109.9	4.6–39	0.1–1.7	7.6–76.8	[34]
1.72–2.98	0.94–4.12	N/A	0.06–0.39	0.124–1.4	N/A	7.22–27.5	[35]
5.8–16.50	N/A	0.13–18.31	55–214	47–334	15–47	40–197	[25]
7.2–319.3	0.11–8.68	N/A	67.0–116.0	35.6–94.8	15.4–84.0	50.20–1510.3	[33]
100	2	25	150	50	100	300	WHO

N/A: non-available.

### 3.2 Contamination degree and ecological risk

#### 3.2.1 Soil contamination indices

**Fig. 2:**
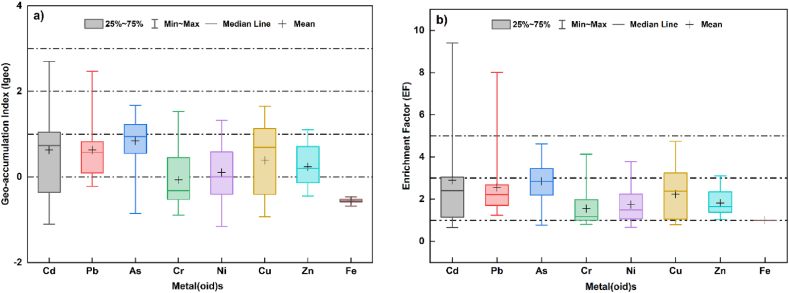
Geo-accumulation index **(a),** and Enrichment factor **(b)** values for the pollutants in the surface soils.

The enrichment factor was computed to distinguish elements derived from human activities and those of natural origin (Jiang et al., 2017). As depicted in Fig. 2, the mean EF values for Cd, Pb, As, Cr, Ni, Cu, and Zn were 2.84, 2.51, 2.80, 1.54, 1.72, 2.20, and 1.79, respectively, with ranges of 0.65–9.40, 1.00–8.01, 0.78–4.62, 0.81–4.13, 0.67–3.78, 0.79–4.74 and 1.00–3.11, respectively. Based on the classification of EF presented in Table 1, most of the examined sites are significantly enriched by the examined metal(oid)s in the studied soils, with EF values higher than one. Significant variation was observed among the studied samples, reflecting that the enrichment of surface soils by TMs is likely to be primarily from anthropogenic inputs.

Amongst the examined pollutants, Pb, Cd, As, and Cu displayed moderate enrichment, which may be linked to the leakage of leachate from the landfill and the use of fertilizers and pesticides. Similar findings were reported previously by Adamcová et al. [36] and Rezapour et al. [37], who found that the leakage from landfills led to significant enrichment of surface soils by Pb and Cd compared to other TMs.

The Geo-accumulation Index (Igeo) was employed to analyze the contamination degree in the investigated area. The box plot diagram (Fig. 2) shows a wide range of calculated values across the sampling sites, with average values decreasing in the following order: As (0.84) > Cd (0.60) > Pb (0.59) > Cu (0.36) > Zn (0.21) > Ni (0.8) > Cr (−0.09) > Fe (−0.58). As, Cd and Pb exhibited the highest pollution, with mean values of 0.84, 0.60, and 0.59 respectively.

Based on the Igeo classification given in Table 1, most of the sampling sites were categorized as uncontaminated to moderately polluted. However, the samples located near the landfill (S1∼S8) were found to be moderately to heavily polluted by Cd, As, and Pb. Similar findings were reported by Taati et al. [38] in their investigation related to agricultural soils in the Arak industrial area (Iran), where surface soils showed low Igeo values (less than 0) for Cu, Zn, and Ni, indicating non-contamination. On the other hand, As and Cd exhibited a moderate impact with average Igeo values of 2.7 and 1.61, respectively.

Shakil et al. [39] and Adelopo et al. [40] also reported significant Igeo values of Cd in surface soils around landfills and dumping sites. It is important to note that exposure to elevated amount of Cd could be harmful to the kidneys, lungs, and reproductive systems of adults, and it may also impair learning, behavior, cognition, and neuromotor skills. Therefore, urgent intervention is required to mitigate the potential risks linked with the accumulation of Cd in this area.

**Fig. 3:**
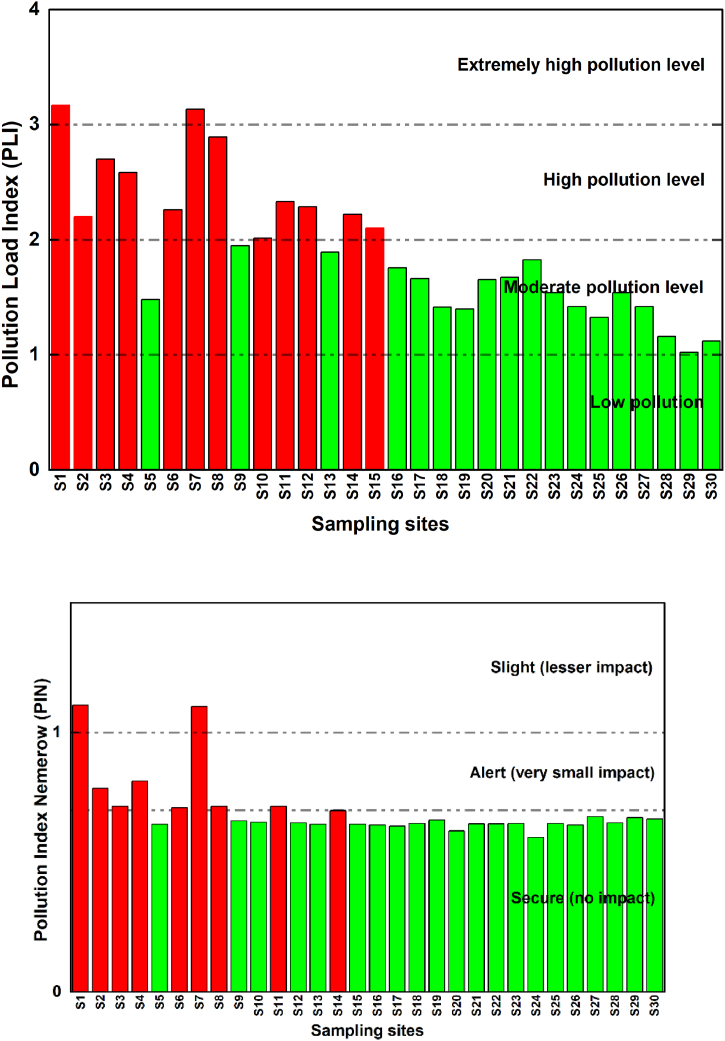
Assessment pollution indices of the studied surface soils; **(a)** Pollution load index, (**b)** Nemerow index method.

The poly-metallic pollution level at each examined soil sample was assessed using the pollution load index, as shown in Fig. 3-a. The obtained values ranged from 1.02 to 3.39, with an average of 1.90. About 40 % of the sampling displayed a PLI greater than 2, indicating a moderate pollution. Specifically, the sample sites, S1∼S4, S6∼S8, S10∼S12 and S14∼S15 were marked as the most polluted compared to other sites, having PLI values ranging from 2.10 to 3.39. These sites showed higher levels of pollution, primarily driven by the accumulation of Cd, Pb, and As.

Similarly, previous studies conducted by Rezapour et al[37] and Essien et al[35] have reported moderate to strong poly-metallic pollution in agricultural soils in Nigeria and Iran, respectively. These elevated pollution levels were attributed to the increased concentrations of toxic pollutants.

However, it is worth noting that the pollution levels in our study area remained below the guidelines set by the World Health Organization (WHO). This is evident from the low values obtained when using the Nemerow index based on the above-cited guidelines. The average PIN of each metal(oid) presented in Fig. 3-b followed the order: Ni (0.51) > Cd (0.44) > Pb (0.25) > Zn (0.16) > Cu (0.13) > As (0.07).

### 3.3 Potential ecological risks

**Fig. 4:**
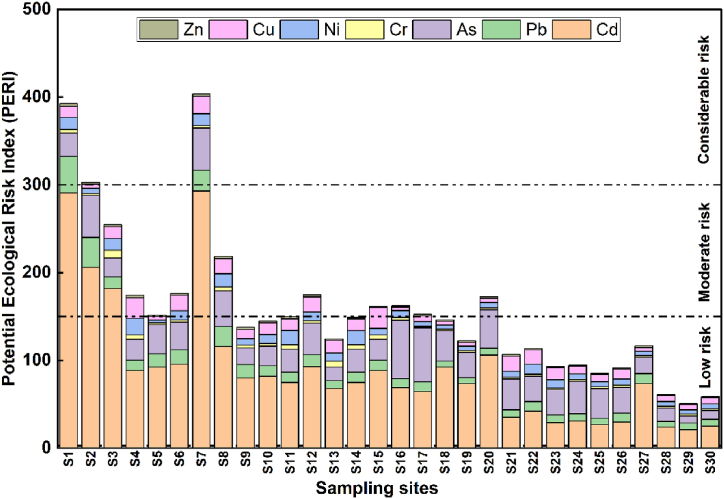
Potential ecological risk index (PERI) values.

The individual ecological risk (Er_i_) and Potential Ecological Risk Index (PERI) values were introduced to evaluate the potential hazard of toxic pollutants on the surrounding environment. This assessment combined the toxicity coefficient and soil geochemical background. The computed values are illustrated in Fig. 4.

The obtained results showed that the Er_i_ of TMs in the surface soils was in the following order: Cd > As > Pb > Cu > Ni > Cr > Zn > Fe. Cu, Cr, Ni, and Zn exhibited a low ecological risk, as their Eri values were below 40 in all examined samples. In contrast, Cd, Pb, and As exhibited elevated ecological risk in almost all samples, emerging as the primary contributors to the PERI. This is ascribed not solely to their prevalence in the soils but also to their higher toxicity factors when compared to other metal(oid)s.

Comprehensive ecological risk varied greatly among the sampling sites, ranging from 50.54 to 411.66. About 36.37 % of the samples had PERI values higher than 150 (Fig. 4), suggesting a moderate ecological risk arising from the accumulation of TMs. Notably, Cd, As, and Pb were the primary contributors to these outcomes, which is in line with previous studies conducted by Varol et al[41], Iqbal et al[42], and Karimian et al[43] related to surface soils exposed to various anthropogenic stressors. The findings indicate that soil contamination may persist in the future if the increase in pollutants continues, particularly Cd, Pb, and As. Therefore, it is crucial to implement measures to mitigate further contamination and protect the environment and human from potential risks.

### 3.4 Source apportionment using PCA, HCA and PMF model

Given the non-normal distribution of the observed data, the correlation between the eight metal(oid)s in topsoils was appraised using Spearman correlation coefficients, as presented in Table S4. The results showed a notable positive correlation among all investigated metal(oid)s, The highly positive correlation among toxic pollutants reveals that they may have the same sources [44]. Therefore, the application of PCA and PMF are of ultimate importance to explain these associations [45].

Principal component analysis (PCA) was introduced to identify the possible sources of pollution in the examined area as depicted in Table 4. Firstly, Bartlett's sphericity test value (p < 0.0001) and Kaiser-Meyer-Olkin (KMO) score (0.657) reflected the accuracy of the data for PCA. The first three PCs contribute to 82 % of the total variance (45.33 % for F1, 22.13 % for F2, and 14.54 % for F3) with an eigenvalue superior to 1 (3.81 for F1, 1.53 for F2, and 1.20 for F3). The initial component, accounts for 47.57 % of the overall cumulative variance and demonstrates a significant correlation with all evaluated pollutants, each having a loading value greater than 0.477. The primary component can be characterized as anthropogenic, confirming that a substantial proportion of the investigated trace metal(oid)s primarily originated from human activities. Meanwhile, PC2 demonstrated positive loadings of Cd, Pb, and As suggesting that they were mainly the result of human activities in the study area as confirmed by the EF values.

**Table 4:** Rotated component for total trace metal (oid)s in examined soils.

	PC1	PC2	PC3	PC4
Cd	**0.823**	**0.417**	0.146	0.191
Pb	**0.765**	**0.484**	0.220	0.028
As	**0.477**	**0.713**	−0.340	−0.165
Cr	**0.579**	−0.596	−0.055	0.502
Ni	**0.796**	−0.464	−0.101	−0.119
Cu	**0.595**	−0.504	−0.313	−0.451
Zn	**0.892**	−0.019	0.056	0.004
Fe	0.132	−0.174	**0.929**	−0.233
Eigenvalue	3.627	1.771	1.163	0.588
% of variance	45.331	22.132	14.540	7.348
Cumulative %	45.331	67.463	82.003	89.351

**Fig. 5:**
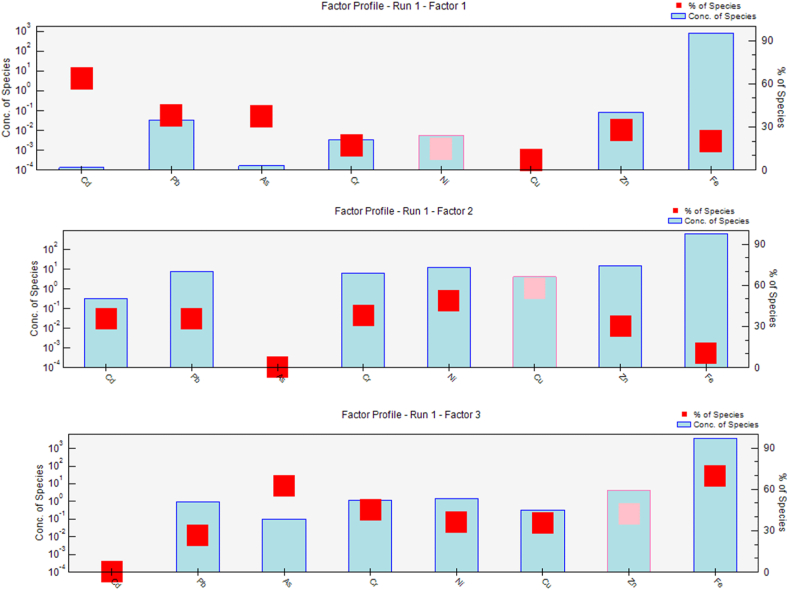
Profiles and contributions percentage of metal (oid)s from the PMF model.

PMF has been used as an effective technique for source apportionment of TMs in previous studies worldwide through the calculation of the contribution of the sources of pollution [7,18,[46], [47], [48]]. Based on the outcomes depicted in Fig. 5, the PMF model parsed out 3 factors. The signal-to-noise (S/N) ratios for the metal (oid)s ranged from 2.4 to 4. Several factors (3–6) were evaluated, and the system was run 20 times to achieve reliable results. Likewise, to the PCA results that showed that the first three-components explain 82 % of the datasets, the best solution from the applied model confined three factors, which ended in the lowest Q value, for which the residuals lied between −1 and +1. Also, the coefficient of determination (r^2^) between the predicted and the observed value was found ranged from 0.762 (Zn) to 0.968 (Cd), indicating a strong correlation among them. Hence, the used PMF model was accurately apportioned the studied toxic pollutants, and the outcomes were reliable.

Factor 1 was predominantly composed of Cd (63.8 %), Pb (37.9 %), and As (37.6 %). The enrichment factor (EF) values of these trace metals (TMs) indicated a significant accumulation in the topsoil. Additionally, they exhibited substantial coefficients of variation, implying that their concentrations were primarily influenced by human activities. Previous papers have shown that these pollutants originate from anthropogenic sources, including landfilling, manure application, fertilizer usage, and traffic emissions [7,44,49]. Therefore, the first factor can be attributed to various anthropogenic activities.

The prominent trace metals (TMs) within factor 2 included Cu (57.5 %), Ni (49 %), Cr (37.7 %), Cd (35.7 %), Pb (35.4 %), and Zn (30.3 %). Notably, a substantial positive correlation was detected among these elements, with their concentrations surpassing their respective geochemical backgrounds. This suggests a common anthropogenic origin [44] and implies that human activities such as landfilling, traffic emissions, and agricultural practices contributed significantly in the accumulation of these toxic pollutants.

Factor 3 was distinguished by the prevalence of Fe (69.7 %). Notably, Fe displayed the highest values among the analyzed metals. However, its concentration in surface soils closely approximated its geochemical background and exhibited a low coefficient of variation in contrast to other metals with the lowest enrichment factor values. Hence, it can be inferred that parent materials predominantly contribute to the presence of iron in the soil.

### 3.5 Human Health Risk Assessment (HHRA)

#### 3.5.1 Deterministic non-carcinogenic health risk

Non-carcinogenic risk for adults and children living in the examined area considering ingestion, dermal, and inhalation contamination pathways are depicted in Table 5. As clearly shown, non-carcinogenic impact on human health expressed in terms of HQs, HIs, and THIs values for ingestion pathway were found to be higher in comparison to other considered routes of exposure, which is in line with previous investigations that highlighted that ingestion is the main pathway could pose the highest risk [21,35]. In addition, Pb, Cd, and As exhibited the highest hazard index (HI) in regard to other metal (oid)s for both age groups owing to their elevated contents in surface soils and lower reference dose (R*f*D). It should be pointed out that it was found that for all trace metal (oid)s, HQs, HIs, and THs for children were higher than those for adults for all exposure pathways. For example, the average THI was nearly ten times higher for children (THI = 0.425) than for adults (THI = 0.043). Because children were the most vulnerable group to environmental contaminants due to their lower sensitivity and immunology [[49], [50], [51]]. Nevertheless, up to this point, the hazard index has consistently remained below the acceptable threshold of 1 in all scrutinized soil samples, this implies that exposure to toxic pollutants is still considered acceptable for individuals residing in the investigated area. However, the continuous accumulation of pollutants concentration could initiate a whole chain of adverse dangerous effects on human health in the future, especially since other pollutants are not measured in the present investigation (e.g., Hg and Co) which also could bring a non-negligible influence on human health.

**Table 5:** Deterministic non-carcinogenic human health risks.

Metal (oid)s	level	conce (mg⋅kg−1)	Children								HI Total Pahway	Adults						HI Total Pathway
			Oral ingestion			Dermal exposure			Inhalation			Oral ingestion		Dermal exposure		Inhalation		
			ADD	HQ		ADD	HQ		ADD	HQ		ADD	HQ	ADD	HQ	ADD	HQ	
**Pb (mg⋅kg−1)**	Min	12.24	1.56E-04	4.47E-02		4.38E-07	8.34E-04		4.20E-09	1.19E-06	4.55E-02	1.68E-05	4.79E-03	6.80E-08	1.30E-04	2.46E-09	7.00E-07	4.92E-03
	Max	78.97	1.01E-03	2.88E-01		2.83E-06	5.38E-03		2.71E-08	7.70E-06	2.94E-01	1.08E-04	3.09E-02	4.39E-07	8.37E-04	1.59E-08	4.52E-06	3.17E-02
	Mean	24.86	3.18E-04	9.08E-02		8.90E-07	1.70E-03		8.53E-09	2.42E-06	9.25E-02	3.41E-05	9.73E-03	1.38E-07	2.63E-04	5.01E-09	1.42E-06	1.00E-02
**Cu (mg⋅kg−1)**	Min	2.76	3.53E-05	8.82E-04		9.87E-08	8.23E-06		9.46E-10	2.37E-08	8.90E-04	3.78E-06	9.45E-05	1.53E-08	1.28E-06	5.56E-10	1.39E-08	9.57E-05
	Max	16.48	2.11E-04	5.27E-03		5.90E-07	4.92E-05		5.66E-09	1.41E-07	5.32E-03	2.26E-05	5.64E-04	9.17E-08	7.64E-06	3.32E-09	8.30E-08	5.72E-04
	Mean	4.10	5.24E-05	1.31E-03		1.47E-07	1.22E-05		1.41E-09	3.52E-08	1.32E-03	5.62E-06	1.40E-04	2.28E-08	1.90E-06	8.26E-10	2.06E-08	1.42E-04
**Cr (mg⋅kg−1)**	Min	9.71	1.24E-04	4.14E-02		3.48E-07	5.80E-03		3.33E-09	1.17E-04	4.73E-02	1.33E-05	4.44E-03	5.40E-08	9.00E-04	1.96E-09	6.84E-05	5.40E-03
	Max	51.90	6.64E-04	2.21E-01		1.86E-06	3.10E-02		1.78E-08	6.23E-04	2.53E-01	7.11E-05	2.37E-02	2.89E-07	4.81E-03	1.05E-08	3.66E-04	2.89E-02
	Mean	19.10	2.44E-04	8.14E-02		6.84E-07	1.14E-02		6.55E-09	2.29E-04	9.30E-02	2.62E-05	8.72E-03	1.06E-07	1.77E-03	3.85E-09	1.35E-04	1.06E-02
**Zn (mg⋅kg−1)**	Min	29.20	3.73E-04	1.24E-03		1.05E-06	1.74E-05		1.00E-08	3.34E-08	1.26E-03	4.00E-05	1.33E-04	1.62E-07	2.71E-06	5.88E-09	1.96E-08	1.36E-04
	Max	85.41	1.09E-03	3.64E-03		3.06E-06	5.10E-05		2.93E-08	9.77E-08	3.69E-03	1.17E-04	3.90E-04	4.75E-07	7.92E-06	1.72E-08	5.74E-08	3.98E-04
	Mean	49.13	6.28E-04	2.09E-03		1.76E-06	2.93E-05		1.69E-08	5.62E-08	2.12E-03	6.73E-05	2.24E-04	2.73E-07	4.55E-06	9.90E-09	3.30E-08	2.29E-04
**Ni (mg⋅kg−1)**	Min	9.64	1.23E-04	6.16E-03		3.45E-07	6.39E-05		3.31E-09	3.68E-05	6.26E-03	1.32E-05	6.60E-04	5.36E-08	9.93E-06	1.94E-09	2.16E-05	6.92E-04
	Max	53.73	6.87E-04	3.43E-02		1.92E-06	3.56E-04		1.84E-08	2.05E-04	3.49E-02	7.36E-05	3.68E-03	2.99E-07	5.53E-05	1.08E-08	1.20E-04	3.86E-03
	Mean	25.44	3.25E-04	1.63E-02		9.11E-07	1.69E-04		8.73E-09	9.70E-05	1.65E-02	3.48E-05	1.74E-03	1.41E-07	2.62E-05	5.12E-09	5.69E-05	1.83E-03
**Cd (mg⋅kg−1)**	Min	0.21	2.68E-06	2.68E-03		7.52E-09	7.52E-04		7.21E-11	7.21E-06	3.44E-03	2.88E-07	2.88E-04	1.17E-09	1.17E-04	4.23E-11	4.23E-06	4.09E-04
	Max	2.93	3.75E-05	3.75E-02		1.05E-07	1.05E-02		1.01E-09	1.01E-04	4.81E-02	4.01E-06	4.01E-03	1.63E-08	1.63E-03	5.90E-10	5.90E-05	5.70E-03
	Mean	0.89	1.14E-05	1.14E-02		3.18E-08	3.18E-03		3.05E-10	3.05E-05	1.46E-02	1.22E-06	1.22E-03	4.94E-09	4.94E-04	1.79E-10	1.79E-05	1.73E-03
**As (mg⋅kg−1)**	Min	0.52	6.65E-06	2.22E-02		1.86E-08	1.51E-04		1.78E-10	5.95E-07	2.23E-02	7.12E-07	2.37E-03	2.89E-09	2.35E-05	1.05E-10	3.49E-07	2.40E-03
	Max	2.99	3.82E-05	1.27E-01		1.07E-07	8.70E-04		1.03E-09	3.42E-06	1.28E-01	4.09E-06	1.36E-02	1.66E-08	1.35E-04	6.02E-10	2.01E-06	1.38E-02
	Mean	1.81	2.31E-05	7.71E-02		6.48E-08	5.27E-04		6.21E-10	2.07E-06	7.77E-02	2.48E-06	8.26E-03	1.01E-08	8.18E-05	3.65E-10	1.22E-06	8.35E-03
**Fe (mg⋅kg−1)**	Min	5671.50	7.25E-02	1.04E-01		2.03E-04	4.51E-03		1.95E-06	8.85E-03	1.17E-01	7.77E-03	1.11E-02	3.15E-05	7.01E-04	1.14E-06	5.19E-03	1.70E-02
	Max	6583.00	8.42E-02	1.20E-01		2.36E-04	5.24E-03		2.26E-06	1.03E-02	1.36E-01	9.02E-03	1.29E-02	3.66E-05	8.14E-04	1.33E-06	6.03E-03	1.97E-02
	Mean	6183.32	7.91E-02	1.13E-01		2.21E-04	4.92E-03		2.12E-06	9.64E-03	1.28E-01	8.47E-03	1.21E-02	3.44E-05	7.64E-04	1.25E-06	5.66E-03	1.85E-02
																		
THI for min. values (TMs)				2.23E-01			1.21E-02			9.01E-03	**2.44E-01**		2.15E-02		1.86E-03		5.29E-03	**2.86E-02**
THI for max. values (TMs)				8.38 E−01			5.34E-02			1.12E-02	**9.03E-01**		7.61E-02		8.16E-03		6.58E-03	**9.09E-02**
THI for mean. Values (TMs)				3.93E-01			2.19E-02			1.00E-02	**4.25E-01**		3.39E-02		3.32E-03		5.87E-03	**4.31E-02**

Note: E is the abbreviation of exponent, which means the index based on 10.

#### 3.5.2 Deterministic carcinogenic human health risk

The deterministic carcinogenic risk due to the accumulation of pollutants in surface soils was calculated considering the multi-pathway exposure to Pb, Cr, As, Ni, and Cd for which the slope factor (SF) data was accessible following USEPA guidelines [26]. Based on the findings depicted in Table 6, CR and TCR risks for both age groups (children and adults) pointed out that ingestion was found as the principal pathway for carcinogenic risk. Concerning the carcinogenetic impact, the average calculated total cancer risk (TCR) values were 7.60E-04 and 8.32E-05 for children and adults. Thus, the carcinogenic was higher than the acceptable limit of 10^−6^ for both age groups. Furthermore, children are more vulnerable to cancer risks, as their average TCR was higher than the threshold value of 10^−4^, which reflects that exposure to TMs could bring considerable cancer risk. It is pointed out that these findings were found much higher in comparison to other previous papers performed in Turkey [51] and Saudi Arabia [52] and in the same range as what was reported in India [27].

**Table 6:** Deterministic carcinogenic human health risks.

Metal (oid)s	level	conce (mg⋅kg−1)	Children							Adults						
			Oral ingestion		Dermal exposure		Inhalation		TCR total pathways	Oral ingestion		Dermal exposure		Inhalation		TCR total pathways
Pb (mg⋅kg−1)	Min	12.24	1.33E-06		3.72E-09		3.71E-07		1.70E-06	1.42E-07		5.78E-10		1.04E-10		1.24E-06
	Max	78.97	8.58E-06		2.40E-08		2.25E-07		8.83E-06	9.19E-07		3.73E-09		6.68E-10		9.24E-07
	Mean	24.86	2.70E-06		7.57E-09		2.64E-06		5.35E-06	2.89E-07		1.18E-09		2.10E-10		2.91E-07
Cr (mg⋅kg−1)	Min	9.71	6.21E-05		6.96E-07		1.40E-07		6.29E-05	6.65E-06		1.08E-07		8.22E-08		6.84E-06
	Max	51.90	3.32E-04		3.72E-06		7.48E-07		3.36E-04	3.55E-05		5.77E-07		4.39E-07		3.66E-05
	Mean	19.10	1.22E-04		1.37E-06		2.75E-07		1.24E-04	1.31E-05		2.12E-07		1.62E-07		1.35E-05
Ni (mg⋅kg−1)	Min	9.64	2.10E-04		1.47E-05		2.78E-09		2.24E-04	2.25E-05		2.28E-06		1.63E-09		2.47E-05
	Max	53.73	1.17E-03		8.17E-05		1.55E-08		1.25E-03	1.25E-04		1.27E-05		9.09E-09		1.38E-04
	Mean	25.44	5.53E-04		3.87E-05		7.33E-09		5.92E-04	5.92E-05		6.01E-06		4.30E-09		6.53E-05
Cd (mg⋅kg−1)	Min	0.21	1.02E-06		2.86E-09		4.54E-10		1.02E-06	1.09E-07		4.44E-10		2.67E-10		1.10E-07
	Max	2.93	1.42E-05		3.99E-08		6.33E-09		1.43E-05	1.53E-06		6.19E-09		3.72E-09		1.54E-06
	Mean	0.89	4.32E-06		1.21E-08		1.92E-09		4.33E-06	4.63E-07		1.88E-09		1.13E-09		4.66E-07
As (mg⋅kg−1)	Min	0.52	9.97E-06		6.81E-08		2.69E-09		1.00E-05	1.07E-06		1.06E-08		1.58E-09		1.08E-06
	Max	2.99	5.73E-05		3.92E-07		1.55E-08		5.77E-05	6.14E-06		6.08E-08		9.09E-09		6.21E-06
	Mean	1.81	3.47E-05		2.37E-07		9.38E-09		3.50E-05	3.72E-06		3.68E-08		5.51E-09		3.76E-06
																
Cumulative carcinogenic risk for min. values			2.84E-04		1.54E-05		5.17E-07		**3.00E-04**	3.04E-05		2.40E-06		8.58E-08		**3.40E-05**
Cumulative carcinogenic risk for max values			1.58E-03		8.59E-05		1.01E-06		**1.67E-03**	1.69E-04		1.33E-05		4.62E-07		**1.83E-04**
Cumulative carcinogenic risk for mean. values			7.17E-04		4.03E-05		2.94E-06		**7.60E-04**	7.68E-05		6.27E-06		1.73E-07		**8.32E-05**

Note: E is the abbreviation of exponent, which means the index based on 10.

#### 3.5.3 The Monte Carlo simulation (MCS)

**Fig. 6:**
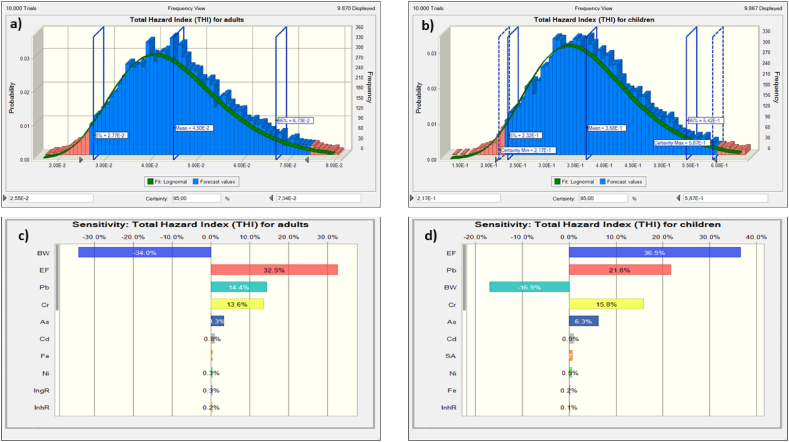
Simulated THI: a) Adults; b) Children; c) Sensitivity diagram for adults; d) Sensitivity diagram for children.

**Fig. 7:**
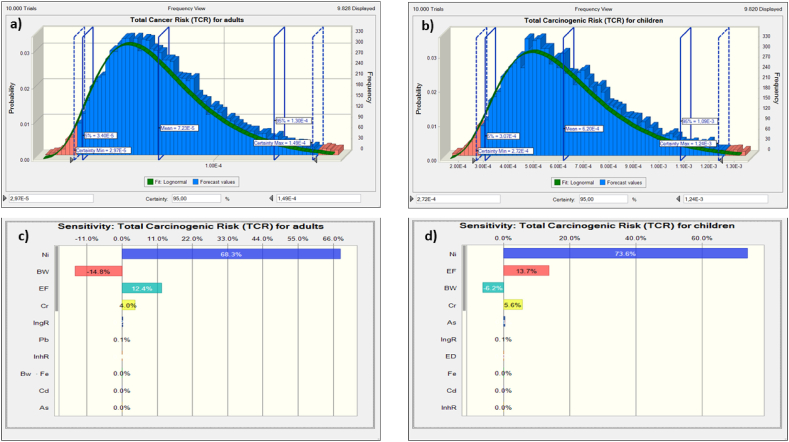
Simulated TCR: a) TCR for adults; b) TCR for children; c) sensitivity diagram for adults; d) sensitivity diagram for children.

Several reasons could lead to an overdetermination or underdetermination of the above-calculated human health risk including body weight, metal (oid)s concentration, exposure duration, exposure frequency, etc. To overcome these limitations Montecarlo simulation (what-if-analysis) was introduced in the present study. Histograms and tornado sensitivity diagrams were used to simulate THI and TCR as depicted in Fig. 6 and **7**. The probability estimation showed that the 50th percentile values obtained from the probabilistic approach closely matched the NCR values calculated using the deterministic method. Comparing the average values for children (0.425) and adults (0.368) (Table 5) with the 50th percentile of risk distribution from the probabilistic approach (0.368 for children and 0.045 for adults), a strong similarity was observed. Moreover, the analysis revealed that children had a higher probability of experiencing non-carcinogenic adverse health effects compared to adults, with a 95th percentile value of 0.542, as opposed to 0.067 for adults. This observation aligns with findings from previous research studies [28,53], which also concluded that children were the most susceptible segment within the exposed population.

Furthermore, the probability distribution of total carcinogenic risks depicted in Fig. 7 (a-b) highlighted that both children and adults faced significant potential risks due to the accumulation of TMs in surface soils. The 5th percentile and mean values of TCR for children ranged between 3.07E-4 and 6.20E-4, while for adults, they ranged between 3.40E-5 and 7.23E-5. These values exceeded the acceptable threshold of 1E-06 by approximately 307 and 34 times for children and adults, respectively. This indicates that nearly 95 % of the population living in the study area could potentially face cancer risks if appropriate corrective measures are not implemented promptly, particularly for children.

#### 3.5.4 Sensitivity and uncertainty analysis

Analysis of sensitivity was also used in the current paper to highlight the most influential parameters in the calculated THI and TCR resulting from long-term exposure to TMs. The results are presented as tornado plots in Fig. 6 (c-d) and Fig. 7 (c-d).

The outcomes indicated that exposure frequency was the significant factor, contributing the highest positive variability of 32.5 % for adults and 36.5 % for children. Following EF, Pb, Cr, As, and Cd were also influential parameters for both adults and children, with varying positive contributions: 14.4 %, 13.6 %, 8.3 %, and 0.8 % for adults, and 21.8 %, 15.8 %, 6.3 %, and 0.9 % for children, respectively.

On the other hand, body weight (BW) was identified as the most insignificant parameter in the estimated THI, exhibiting a total variability of −34 % for adults and −16.90 % for children. In other words, an increase in body weight can significantly reduce potential health risks associated with TMs exposure [54].

The tornado plots of carcinogenic risk showed that EF, Ni, and Cr were identified as the most responsive risk indicators, contributing 12.4 %, 68.3 %, and 4 % to the overall risk for adults, and 13.7 %, 73.6 %, and 5.6 % for children, respectively. These results highlight the significant impact of these metal (oid)s on the potential carcinogenic risk to human health, especially Ni, which had a significant contribution among the studied pollutants, emphasizing the importance of strict control measures for this particular contaminant.

#### 3.5.5 Limitations of this study

This study presents several limitations that should be taken into consideration. Specifically, the analysis focused solely on the total concentration of each metal (oid). Nevertheless, it is well-known that the toxicity of most pollutants is heavily linked to their chemical forms and valence state. As an example, inorganic arsenic and Cr VI are regarded as the toxic forms of arsenic and chromium, respectively.

Given these limitations, it is crucial to exercise caution when interpreting the results of this risk assessment. The exclusion of specific forms and chemical species of trace metals may not fully capture the actual health risks associated with each metal (loid) present in the soil. In future studies, a more comprehensive approach should be employed, incorporating the consideration of various metal species, to provide a more accurate evaluation of potential health hazards for both human and environmental health.

## 4 Conclusion

In this study, we employed comprehensive approach combining PCA, PMF, ecological risk, pollution indices, probabilistic and deterministic human health risk to thoroughly evaluate the source contribution of each pollution source, ecological risk, and human risks linked with the accumulation of metal (oid)s in surface soils of an agricultural area experiencing higher anthropogenic stress. The results unveiled that both landfilling activities and intensive agriculture played a substantial role in the amount of trace metal (oid)s in the investigated area. The majority of the metal (oid)s surpassed their respective background values, signifying a moderate level of enrichment and potential ecological risk. The evaluation of non-carcinogenic and carcinogenic risks suggested that human beings are confronted with a high likelihood of experiencing adverse health influences. The outcomes strongly suggest that intensive agricultural practices, landfilling activities, and traffic emissions are major contributors to the spread of pollutants. By integrating multiple assessment methods, this investigation provides a comprehensive understanding of the environmental and health risks posed by the accumulation of metal (oid)s. It highlights the need for effective and immediate interventions to safeguard human health and the adjacent environment in the affected area.

## Ethical approval

Authors state that the research was conducted according to ethical standards.

## Consent to participate

Not applicable.

## Consent to publish

Not applicable.

## Funding

This research received no external funding.

## Data availability

Data associated with the study has been deposited into a publicly available repository.

## CRediT authorship contribution statement

**Hamza El Fadili:** Writing – review & editing, Writing – original draft, Visualization, Validation, Supervision, Software, Resources, Project administration, Methodology, Investigation, Formal analysis, Data curation, Conceptualization. **Mohammed Ben Ali:** Writing – review & editing, Writing – original draft, Validation, Supervision, Methodology, Investigation, Data curation, Conceptualization. **Md Naimur Rahman:** Writing – review & editing, Writing – original draft, Validation, Investigation. **Mohammed El Mahi:** Writing – review & editing, Writing – original draft, Validation. **El Mostapha Lotfi:** Writing – review & editing, Writing – original draft, Validation. **Sami Louki:** Writing – review & editing, Validation.

## Declaration of competing interest

The authors declare that they have no known competing financial interests or personal relationships that could have appeared to influence the work reported in this paper.

## References

[1] El Fadili H., Ben Ali M., Safhi A. el M., El Mahi M., Aziz A., Lotfi E.M. (2023). Effects of encapsulating cellulose acetate microfibers on the mechanical, thermal and environmental properties of geopolymers: A new solution to mitigate the cigarettes pollution. J. Build. Eng..

[2] Kaza S., Yao L.C., Bhada-Tata P., Van Woerden F. (2018).

[3] El Fadili H., Ben Ali M., El M., Nabil M., Lotfi M., Labjar N., Ibn S., Abdelhamid E. (2023). Determination of properties and environmental impact due to the inclusion of cigarette fibers in mortar: a new solution to mitigate the CB pollution. Environ. Sci. Pollut. Res..

[4] Ben Ali M., El Fadili H., El Mahi M., Lotfi E.M., Fannakh A., Chahine A. (2023). Geochemistry pollution status and ecotoxicological risk assessment of heavy metal(oid)s in soil influenced by co-landfilling of MSW and sewage sludge, Morocco. Environ. Nanotechnol. Monit. Manag..

[5] Law H.J., Ross D.E. (2019). International Solid Waste Association's “closing dumpsites” initiative: status of progress. Waste Manag. Res..

[6] Varol M. (2020). Environmental, ecological and health risks of trace metals in sediments of a large reservoir on the Euphrates River (Turkey). Environ. Res..

[7] Huang C.C., Cai L.M., Xu Y.H., Wen H.H., Jie L., Hu G.C., Chen L.G., Wang H.Z., Xu X.B., Mei J.X. (2022). Quantitative analysis of ecological risk and human health risk of potentially toxic elements in farmland soil using the PMF model. Land Degrad. Dev..

[8] Bouzekri S., Laarbi M., Hachimi E., Touach N., El H., El M., Mostapha E. (2019). Journal of African Earth Sciences The study of metal (As , Cd , Pb , Zn and Cu) contamination in super fi cial stream sediments around of Zaida mine (High Moulouya-Morocco).

[9] Varol M., Gündüz K., Ras M. (2021). Pollution status , potential sources and health risk assessment of arsenic and trace metals in agricultural soils : a case study in Malatya province. Turkey.

[10] Hussein M., Yoneda K., Mohd-Zaki Z., Amir A., Othman N. (2021). Heavy metals in leachate, impacted soils and natural soils of different landfills in Malaysia: an alarming threat. Chemosphere.

[11] Adelopo A.O., Haris P.I., Alo B.I., Huddersman K., Jenkins R.O. (2018). Multivariate analysis of the effects of age, particle size and landfill depth on heavy metals pollution content of closed and active landfill precursors. Waste Manag..

[12] Wang S., Han Z., Wang J., He X., Zhou Z., Hu X. (2022). Environmental risk assessment and factors influencing heavy metal concentrations in the soil of municipal solid waste landfills. Waste Manag..

[13] Fei X., Lou Z., Xiao R., Ren Z., Lv X. (2020). Contamination assessment and source apportionment of heavy metals in agricultural soil through the synthesis of PMF and GeogDetector models. Sci. Total Environ..

[14] Jiang H.H., Cai L.M., Wen H.H., Hu G.C., Chen L.G., Luo J. (2020). An integrated approach to quantifying ecological and human health risks from different sources of soil heavy metals. Sci. Total Environ..

[15] Varol M., Gündüz K., Sünbül M.R. (2021). Pollution status, potential sources and health risk assessment of arsenic and trace metals in agricultural soils: a case study in Malatya province, Turkey. Environ. Res..

[16] Thi H., Nhien H., Giao N.T. (2022). Science of the Total Environment Assessment of pollution levels and ecological potential risk of the soil in fl uenced by land fi lling in a Vietnamese Mekong Delta province. Sci. Total Environ..

[17] Guan Q., Wang F., Xu C., Pan N., Lin J., Zhao R., Yang Y., Luo H. (2018). Source apportionment of heavy metals in agricultural soil based on PMF: a case study in Hexi Corridor, northwest China. Chemosphere.

[18] Gu X., Wang Z., Wang J., Ouyang W., Wang B., Xin M., Lian M., Lu S., Lin C., He M., Liu X. (2022). Sources, trophodynamics, contamination and risk assessment of toxic metals in a coastal ecosystem by using a receptor model and Monte Carlo simulation. J. Hazard Mater..

[19] Khodadadi N., Amini A., Dehbandi R. (2022). Contamination, probabilistic health risk assessment and quantitative source apportionment of potentially toxic metals (PTMs) in street dust of a highly developed city in north of Iran. Environ. Res..

[20] Hossain Bhuiyan M.A., Chandra Karmaker S., Bodrud-Doza M., Rakib M.A., Saha B.B. (2021). Enrichment, sources and ecological risk mapping of heavy metals in agricultural soils of dhaka district employing SOM, PMF and GIS methods. Chemosphere.

[21] El Fadili H., Ben Ali M., Touach N., El Mahi M., Lotfi E.M. (2022). Ecotoxicological and pre-remedial risk assessment of heavy metals in municipal solid wastes dumpsite impacted soil in Morocco. Environ. Nanotechnol. Monit. Manag..

[22] El Fadili H., Ben Ali M., El Mahi M., Cooray A.T., Mostapha Lotfi E. (2022). A comprehensive health risk assessment and groundwater quality for irrigation and drinking purposes around municipal solid waste sanitary landfill: A case study in Morocco. Environ. Nanotechnol. Monit. Manag..

[23] Bouzekri S., Fadili H. El, Laarabi M., Hachimi E., Mahi M. El, Lotfi E.M. (2019). Assessment of trace metals contamination in sediment and surface water of quarry lakes from the abandoned Pb. Environ. Dev. Sustain..

[24] AFNOR, French standards association, Rocks-Tests Determ (2000).

[25] Varol M., Sünbül M.R., Aytop H., Yılmaz C.H. (2020). Environmental, ecological and health risks of trace elements, and their sources in soils of Harran Plain, Turkey. Chemosphere.

[26] Usepa M. (2005). Risk Assess. Forum US Environ.

[27] Pirsaheb M., Hadei M., Sharafi K. (2021). Human health risk assessment by Monte Carlo simulation method for heavy metals of commonly consumed cereals in Iran- Uncertainty and sensitivity analysis. J. Food Compos. Anal..

[28] Rajasekhar B., Nambi I.M., Govindarajan S.K. (2018). Human health risk assessment of ground water contaminated with petroleum PAHs using Monte Carlo simulations: a case study of an Indian metropolitan city. J. Environ. Manag..

[29] Li X., Liu H., Meng W., Liu N., Wu P. (2022). Accumulation and source apportionment of heavy metal(loid)s in agricultural soils based on GIS, SOM and PMF: a case study in superposition areas of geochemical anomalies and zinc smelting, southwest China. Process Saf. Environ. Protect..

[30] Islam M., Ahmed M., Habibullah-Al-Mamun M., Raknuzzaman M. (2015). Trace elements in different land use soils of Bangladesh and potential ecological risk. Environ. Monit. Assess..

[31] Kumar P., Dipti, Kumar S., Singh R.P. (2022). Severe contamination of carcinogenic heavy metals and metalloid in agroecosystems and their associated health risk assessment. Environ. Pollut..

[32] Liang Q., Tian K., Li L., He Y., Zhao T., Liu B., Wu Q. (2022). Ecotoxicology and Environmental Safety Ecological and human health risk assessment of heavy metals based on their source apportionment in cropland soils around an e-waste dismantling site , Southeast China, Ecotoxicol. Environ. Saf..

[33] Esmaeili A., Moore F., Keshavarzi B., Jaafarzadeh N., Kermani M. (2014). Catena A geochemical survey of heavy metals in agricultural and background soils of the Isfahan industrial zone , Iran. Catena.

[34] Aydi A. (2015). Assessment of heavy metal contamination risk in soils of landfill of Bizerte (Tunisia) with a focus on application of pollution indicators. Environ. Earth Sci..

[35] Essien J.P., Inam E.D., Ikpe D.I., Udo G.E., Benson N.U. (2019).

[36] Adamcová D., Radziemska M., Ridošková A., Bartoň S., Pelcová P., Elbl J., Kynický J., Brtnický M., Vaverková M.D. (2017). Environmental assessment of the effects of a municipal landfill on the content and distribution of heavy metals in Tanacetum vulgare L. Chemosphere.

[37] Rezapour S., Samadi A., Kalavrouziotis I.K., Ghaemian N. (2018). Impact of the uncontrolled leakage of leachate from a municipal solid waste landfill on soil in a cultivated-calcareous environment. Waste Manag..

[38] Taati A., Salehi M.H., Mohammadi J., Mohajer R., Díez S. (2020). Pollution assessment and spatial distribution of trace elements in soils of Arak industrial area, Iran: Implications for human health. Environ. Res..

[39] Shakil S., Nawaz K., Sadef Y. (2023). Evaluation and environmental risk assessment of heavy metals in the soil released from e ‑ waste management activities in Lahore, Pakistan. Environ. Monit. Assess..

[40] Adelopo A.O., Haris P.I., Alo B.I., Huddersman K., Jenkins R.O. (2018). Multivariate analysis of the effects of age, particle size and landfill depth on heavy metals pollution content of closed and active landfill precursors. Waste Manag..

[41] Varol M., Gündüz K., Sünbül M.R. (2021). Pollution status, potential sources and health risk assessment of arsenic and trace metals in agricultural soils: a case study in Malatya province, Turkey. Environ. Res..

[42] Iqbal A., Tabinda A.B., Yasar A. (2019). Human and Ecological Risk Assessment : an International Environmental risk assessment of a young landfill site and its vicinity for possible human exposure. Hum. Ecol. Risk Assess. An Int. J..

[43] Karimian S., Shekoohiyan S., Moussavi G. (2021). Health and ecological risk assessment and simulation of heavy metal-contaminated soil of Tehran landfill. RSC Adv..

[44] Jiang H.H., Cai L.M., Hu G.C., Wen H.H., Luo J., Xu H.Q., Chen L.G. (2021). An integrated exploration on health risk assessment quantification of potentially hazardous elements in soils from the perspective of sources. Ecotoxicol. Environ. Saf..

[45] Kapelewska J., Kotowska U., Karpińska J., Astel A., Zieliński P., Suchta J., Algrzym K. (2019). Water pollution indicators and chemometric expertise for the assessment of the impact of municipal solid waste landfills on groundwater located in their area. Chem. Eng. J..

[46] El Ouaty O., El M’rini A., Nachite D., Marrocchino E., Marin E., Rodella I. (2022). Assessment of the heavy metal sources and concentrations in the Nador Lagoon sediment, Northeast-Morocco. Ocean Coast Manag..

[47] Lei M., Li K., Guo G., Ju T. (2022). Source-specific health risks apportionment of soil potential toxicity elements combining multiple receptor models with Monte Carlo simulation. Sci. Total Environ..

[48] Yang J., Sun Y., Wang Z., Gong J., Gao J., Tang S., Ma S., Duan Z. (2022). Heavy metal pollution in agricultural soils of a typical volcanic area: risk assessment and source appointment. Chemosphere.

[49] Wang X., Wang L., Zhang Q., Liang T., Li J., Bruun Hansen H.C., Shaheen S.M., Antoniadis V., Bolan N., Rinklebe J. (2022). Integrated assessment of the impact of land use types on soil pollution by potentially toxic elements and the associated ecological and human health risk. Environ. Pollut..

[50] Mallongi A., Astuti R.D.P., Amiruddin R., Hatta M., Rauf A.U. (2021). Identification source and human health risk assessment of potentially toxic metal in soil samples around Karst watershed of pangkajene, Indonesia. Environ. Nanotechnol. Monit. Manag..

[51] Varol M., Tokatlı C. (2023). Evaluation of the water quality of a highly polluted stream with water quality indices and health risk assessment methods. Chemosphere.

[52] Ali I.H., Siddeeg S.M., Idris A.M., Brima E.I., Khalid A., Ebraheem S.A.M., Arshad M., Ali I.H., Siddeeg S.M., Idris A.M., Brima E.I., Khalid A. (2019). Contamination and human health risk assessment of heavy metals in soil of a municipal solid waste dumpsite in Khamees-Mushait , Saudi Arabia. Toxin Rev..

[53] Yang Q., Zhang L., Wang H., Martín J.D. (2022). Bioavailability and health risk of toxic heavy metals (As, Hg, Pb and Cd) in urban soils: a Monte Carlo simulation approach. Environ. Res..

[54] Pasupuleti S., Singha S.S., Singha S., Kumar S., Singh R., Dhada I. (2022). Groundwater characterization and non-carcinogenic and carcinogenic health risk assessment of nitrate exposure in the Mahanadi River Basin of India. J. Environ. Manag..

