# Supplementary material for: Bioavailability and health risk of pollutants around a controlled landfill in Morocco: Synergistic effects of landfilling and intensive agriculture
Source: Heliyon. 2023 Dec 19;10(1):e23729. doi: 10.1016/j.heliyon.2023.e23729 (PMC10776943; doi:10.1016/j.heliyon.2023.e23729)
Supplement: Multimedia component 1 [file mmc1.docx]

**Supplementary material**

**Bioavailability and Health Risk of pollutants around a controlled landfill in Morocco: Synergistic Effects of landfilling and intensive agriculture**

**Hamza El Fadili^a, *^, Mohammed Ben Ali^a^, Md. Naimur Rahman^b,c^, Mohammed El Mahi^a^, El Mostapha Lotfi^a^, Sami Louki^a^**

^a^ Laboratory of Spectroscopy, Molecular Modeling, Materials, Nanomaterials, Water and Environment, Materials for Environment Team, ENSAM, Mohammed V University in Rabat, Morocco

^b^Center for Archaeological Studies, University of Liberal Arts Bangladesh, Dhaka, Bangladesh.

^c^Department of Development Studies, Daffodil International University, Dhaka – 1216.

*** Corresponding author:** hamza_elfadili2@um5.ac.ma **(H. EL FADILI)**, +212767450512

**Table S1. Values of RfD (mg/kg/day) and CSF (per mg/kg/day) for examined metal(oid)s**

|  | **Cd** | **Cr** | **As** | **Pb** | **Cu** | **Zn** | **Ni** | **Fe** |
| --- | --- | --- | --- | --- | --- | --- | --- | --- |
| **R*f*D for ingestion** | 1.00E-03 | 3.00E-03 | 3.00E-04 | 3.50E-03 | 4.00E-02 | 3.00E-01 | 2.00E-02 | 7.00E-01 |
| **R*f*D for dermal absorption** | 1.00E-05 | 6.00E-05 | 1.23E-04 | 5.25E-04 | 1.20E-02 | 6.00E-02 | 5.40E-03 | 4.50E-02 |
| **R*f*D for inhalation** | 1.00E-05 | 2.86E-05 | 3.00E-04 | 3.50E-03 | 4.00E-02 | 3.00E-01 | 9.00E-05 | 2.20E-04 |
| **CSF for ingestion** | 3.80E+01 | 5.00E-01 | 1.50E+00 | 8.50E-03 | - | - | 8.40E-01 | - |
| **CSF for dermal absorption** | 3.80E+01 | 2.00E+00 | 3.66E+00 | 5.30E-04 | - | - | 8.40E-01 | - |
| **CSF for inhalation** | 6.30E+00 | 4.20E+01 | 1.51E+01 | 8.50E-03 | - | - | 8.40E-01 | - |
|  |  |  |  |  |  |  |  |  |

**Table S2. The values of factors that used in the probabilistic carcinogenic and non-carcinogenic risk assessment**

| **Parameters** | **Symbol** | **Units** | **Children** | **Adults** | **Distribution** | **References** |
| --- | --- | --- | --- | --- | --- | --- |
| **Heavy metal concentration** | Csoil | mg/kg | Csoil ± SD | Csoil ± SD | Log-normal | This study |
| **Ingestion rate** | IngR | mg/day | 200 ± 4 | 100 ± 1.7 | Log-normal | (Fadili et al., 2022) |
| **Inhalation rate** | InhR | m3/day | 7.6 ± 2.39 | 20 ± 1.27 | Log-normal | (Karimian et al., 2021) |
| **Exposure frequency** | EF | day/year | 350 (180, 365) | 350 (180, 365) | Triangular | (Karimian et al., 2021) |
| **Exposure duration** | ED | year | 6 ± 2.39 | 30 ± 2.74 | Log-normal | (Karimian et al., 2021) |
| **Conversion factor** | CF | kg/mg | 1 × 10-6 | 1 × 10-6 | Point | (Fadili et al., 2022) |
| **Skin adherence factor** | AF | mg/cm2/day | 0.2 | 0.07 | Point | (Fadili et al., 2022) |
| **Dermal absorption factor** | ABS | unitless | 1.0×10-3 | 1.0×10-3 | Point | (Fadili et al., 2022) |
| **Exposed skin area** | SA | cm2 | 2800 ± 1171 | 5700 ± 440 | Log-normal | (Karimian et al., 2021) |
| **Particle emission factor** | PE | m3/kg | 1.36 × 109 | 1.36 × 109 | Point | (Fadili et al., 2022) |
| **Average body weight** | BW | kg | 15 ± 1.5 | 70 ± 10.71 | Log-normal | (Karimian et al., 2021) |

Table S3. Metal(oid)s concentration in examined surface soils in the study area

| **Cd** | **Pb** | **As** | **Cr** | **Ni** | **Cu** | **Zn** | **Fe** |
| --- | --- | --- | --- | --- | --- | --- | --- |
| 2.91 | 78.97 | 2.85 | 24.66 | 39.19 | 8.79 | 85.41 | 6281.00 |
| 2.06 | 64.90 | 2.93 | 13.43 | 17.19 | 3.27 | 51.24 | 6139.00 |
| 1.82 | 25.03 | 1.36 | 51.90 | 38.27 | 9.33 | 66.99 | 6339.50 |
| 0.89 | 22.30 | 1.48 | 28.80 | 53.73 | 16.48 | 76.14 | 6014.50 |
| 0.92 | 28.18 | 2.09 | 11.34 | 9.64 | 2.92 | 37.78 | 6118.50 |
| 0.95 | 31.08 | 1.99 | 14.58 | 29.62 | 12.52 | 55.68 | 6583.00 |
| 2.93 | 45.25 | 2.99 | 17.66 | 38.27 | 13.97 | 67.74 | 6289.00 |
| 1.16 | 42.84 | 2.54 | 28.08 | 42.50 | 12.03 | 72.59 | 6420.00 |
| 0.80 | 29.42 | 1.17 | 20.52 | 21.34 | 7.19 | 67.20 | 6160.50 |
| 0.82 | 23.13 | 1.38 | 19.20 | 28.89 | 9.26 | 57.62 | 6079.50 |
| 0.75 | 22.15 | 1.62 | 31.08 | 46.92 | 9.35 | 65.05 | 6317.50 |
| 0.93 | 25.25 | 2.25 | 17.66 | 28.27 | 11.97 | 67.74 | 6000.00 |
| 0.68 | 16.23 | 0.98 | 40.56 | 26.42 | 10.04 | 40.36 | 6025.00 |
| 0.75 | 22.15 | 1.62 | 31.08 | 45.92 | 9.35 | 45.05 | 6271.50 |
| 0.89 | 22.30 | 1.48 | 28.80 | 21.73 | 16.48 | 36.14 | 6010.00 |
| 0.69 | 19.54 | 2.16 | 19.70 | 22.27 | 2.76 | 38.22 | 6060.00 |
| 0.64 | 21.06 | 1.65 | 9.71 | 15.13 | 4.75 | 45.85 | 6072.50 |
| 0.92 | 13.18 | 2.15 | 11.34 | 13.64 | 2.92 | 37.78 | 6172.50 |
| 0.74 | 12.24 | 1.80 | 12.55 | 14.20 | 3.05 | 45.75 | 6316.00 |
| 1.06 | 14.90 | 2.74 | 13.43 | 17.19 | 3.27 | 51.24 | 5819.00 |
| 0.35 | 16.51 | 2.19 | 12.45 | 19.44 | 12.09 | 46.70 | 6151.00 |
| 0.42 | 21.30 | 1.79 | 14.32 | 32.15 | 11.47 | 41.50 | 6072.50 |
| 0.29 | 16.50 | 1.81 | 11.02 | 27.15 | 9.50 | 36.19 | 6160.50 |
| 0.31 | 15.12 | 2.31 | 13.07 | 18.40 | 6.12 | 31.67 | 5671.50 |
| 0.27 | 13.40 | 2.11 | 12.50 | 16.30 | 5.80 | 29.20 | 6236.50 |
| 0.30 | 19.76 | 1.83 | 15.06 | 19.54 | 8.22 | 34.68 | 6121.00 |
| 0.74 | 21.24 | 1.15 | 12.55 | 14.20 | 3.05 | 45.75 | 6431.00 |
| 0.24 | 12.50 | 0.94 | 13.10 | 15.30 | 4.60 | 34.50 | 6268.50 |
| 0.21 | 14.23 | 0.52 | 12.18 | 14.11 | 3.96 | 29.49 | 6486.50 |
| 0.25 | 15.20 | 0.63 | 10.54 | 16.20 | 5.02 | 32.50 | 6412.00 |

Table S4. Spearman Correlation coefficients among variables

|  |  |  |  |  |  |  |  |  |  |
| --- | --- | --- | --- | --- | --- | --- | --- | --- | --- |
|  | Variables | Cd | Pb | As | Cr | Ni | Cu | Zn | Fe |
|  | Cd | **1** | **0.838** | **0.589** | 0.315 | **0.386** | 0.200 | **0.690** | 0.129 |
|  | Pb | **0.838** | **1** | **0.563** | 0.154 | 0.340 | 0.170 | **0.637** | 0.178 |
|  | As | **0.589** | **0.563** | **1** | -0.152 | 0.138 | 0.073 | 0.347 | -0.276 |
|  | Cr | 0.315 | 0.154 | -0.152 | **1** | **0.661** | **0.461** | **0.460** | 0.048 |
|  | Ni | **0.386** | 0.340 | 0.138 | **0.661** | **1** | **0.702** | **0.705** | 0.125 |
|  | Cu | 0.200 | 0.170 | 0.073 | **0.461** | **0.702** | **1** | **0.462** | -0.024 |
|  | Zn | **0.690** | **0.637** | 0.347 | **0.460** | **0.705** | **0.462** | **1** | 0.137 |
|  | Fe | 0.129 | 0.178 | -0.276 | 0.048 | 0.125 | -0.024 | 0.137 | **1** |
|  | *Values in bold are different from 0 with a significance level alpha=0,05* | | | | | | |  |  |

**References**

Fadili, H. El, Ali, M. Ben, Touach, N., Mahi, M. El, & Lotfi, E. M. (2022). Environmental Nanotechnology , Monitoring & Management Ecotoxicological and pre-remedial risk assessment of heavy metals in municipal solid wastes dumpsite impacted soil in morocco. *Environmental Nanotechnology, Monitoring & Management*, *17*(January), 100640. https://doi.org/10.1016/j.enmm.2021.100640

Karimian, S., Shekoohiyan, S., & Moussavi, G. (2021). Health and ecological risk assessment and simulation of heavy metal-contaminated soil of Tehran landfill. *RSC Advances*, *11*(14), 8080–8095. https://doi.org/10.1039/d0ra08833a

**Agglomerative hierarchical clustering (AHC) / Number of clusters = 3:**
